# A Mendelian randomization study between metabolic syndrome and its components with prostate cancer

**DOI:** 10.1038/s41598-024-65310-y

**Published:** 2024-06-21

**Authors:** Long Xia, Xiao-dong Yu, Li Wang, Lin Yang, Er-hao Bao, Ben Wang, Ping-yu Zhu

**Affiliations:** 1 Department of Urology, Affiliated Hospital of North Sichuan Medical College, Nanchong, 637000 China; 2 Department of Urology, The Second Hospital of Lanzhou University, Lanzhou, China

**Keywords:** Prostate cancer, Metabolic syndrome, Mendelian randomization, Genome‐wide association studies, Meta-analysis, Cancer, Diseases, Urology

## Abstract

Previous research has produced inconsistent findings concerning the connection between metabolic syndrome and prostate cancer. It is challenging for observational studies to establish a conclusive causal relationship between the two. However, Mendelian randomization can provide stronger evidence of causality in this context. To examine the causal link between a metabolic composite and its components with prostate cancer, we performed a two-sample Mendelian randomization (MR) study utilizing aggregated data from genome-wide association studies, followed by meta-analyses. In our study, we employed inverse variance weighting as the primary method for MR analysis. Additionally, we assessed potential sources of heterogeneity and horizontal pleiotropy through the Cochran’s Q test and MR-Egger regression. Moreover, we used multivariate MR to determine whether smoking versus alcohol consumption had an effect on the outcomes. We found no causal relationship between metabolic syndrome and its components and prostate cancer(MetS, odds ratio [OR] = 0.95, 95% confidence interval [CI] = 0.738–1.223, *p* = 0.691; TG, [OR] = 1.02, 95%[CI] = 0.96–1.08, *p* = 0.59); HDL, [OR] = 1.02, 95% [CI] = 0.97–1.07, *p* = 0.47; DBP, [OR] = 1.00, 95%[CI] = 0.99–1.01, *p* = 0.87; SBP, [OR] = 1.00, 95%[CI] = 0.99–1.00, *p* = 0.26; FBG [OR] = 0.92, 95%[CI] = 0.81–1.05, *p* = 0.23; WC, [OR] = 0.93, 95%[CI] = 0.84–1.03, *p* = 0.16). Finally, the MVMR confirms that the metabolic syndrome and its components are independent of smoking and alcohol consumption in prostate cancer. We didn’t find significant evidence to determine a causal relationship between the metabolic syndrome and its components and prostate cancer through MR analysis. Further research is necessary to explore the potential pathogenesis between the two diseases.

## Introduction

Prostate cancer (PCa) is a highly prevalent social disease among men, projected to comprise 7% of all newly diagnosed cancers in men worldwide, resulting in over 1.2 million new cases annually^1,2^. The etiology of PCa is attributed to various risk factors, including advanced age, race, family history, and smoking^3^. However, as more research is done on PCa, more and more risk factors are being identified, such as metabolic syndrome. Metabolic syndrome (MetS) encompasses a cluster of metabolic abnormalities, such as high blood pressure, abdominal obesity, dyslipidemia, as well as hyperglycemia^4^. The global prevalence of MetS is consistently on the rise^5^, and imposing a significant socio-economic burden, especially among the elderly^6^. Studies have commenced investigating the potential association between MetS, its components, and PCa. In a case–control investigation conducted by Jesús Gibran Hernández-Pérez et al., it was indicated that metabolic syndrome exhibited a substantial probability of PCa, while altered lipids, hypertension, and a notable lifetime body weight gain corresponded to an elevated PCa risk^7^. However, there are also studies that suggest the opposite conclusion. A prospective cohort study by Aaron J Tande et al. concluded that there is an inverse relationship between the occurrence of PCa and the presence of MetS^8^. Additionally, some findings indicate an independent association between MetS and PCa. The outcomes of the EPICAP case–control study conducted by Céline Lavalette et al. showed no association between MetS and PCa^9^. Furthermore, extensive research on various aspects of PCa has significantly advanced its treatment methodologies. In a comprehensive review by Alessandro Rizzo et al., they examined the feasibility of immunotherapy for PCa and provided a detailed overview of the current status of PCa vaccines and immune checkpoint monoclonal antibodies, aiming to enhance their application in the treatment of this disease^10^. Similarly, a meta-analysis conducted by Veronica Mollica et al.^11^ investigated whether the ECOG PS score affects the survival rate of immunotherapy and the findings indicated that immunotherapy, whether used alone or in combination, is effective in controlling the progression of PCa. In addition to immunotherapy, a study by Matteo Rosellini et al.^12^ found that antibody–drug conjugates (ADCs) can effectively control the clinical activity of PCa, thereby enhancing the efficacy of PCa treatment. Despite the ongoing in-depth research into various aspects of PCa, various studies have yielded divergent idea about the link between MetS and PCa. The strength of utilizing the Mendelian randomization approach in research lies in its utilization of genetic variance as an instrumental variable, effectively mitigating confounding influence and circumventing reverse cause and effect, thereby Improving the robustness of the results^13^. In this investigation, a two-sample Mendelian randomization analysis was employed to investigate the causal impact of metabolic syndrome and its constituents on PCa.

## Methods

### Study design overview

**Figure 1:**
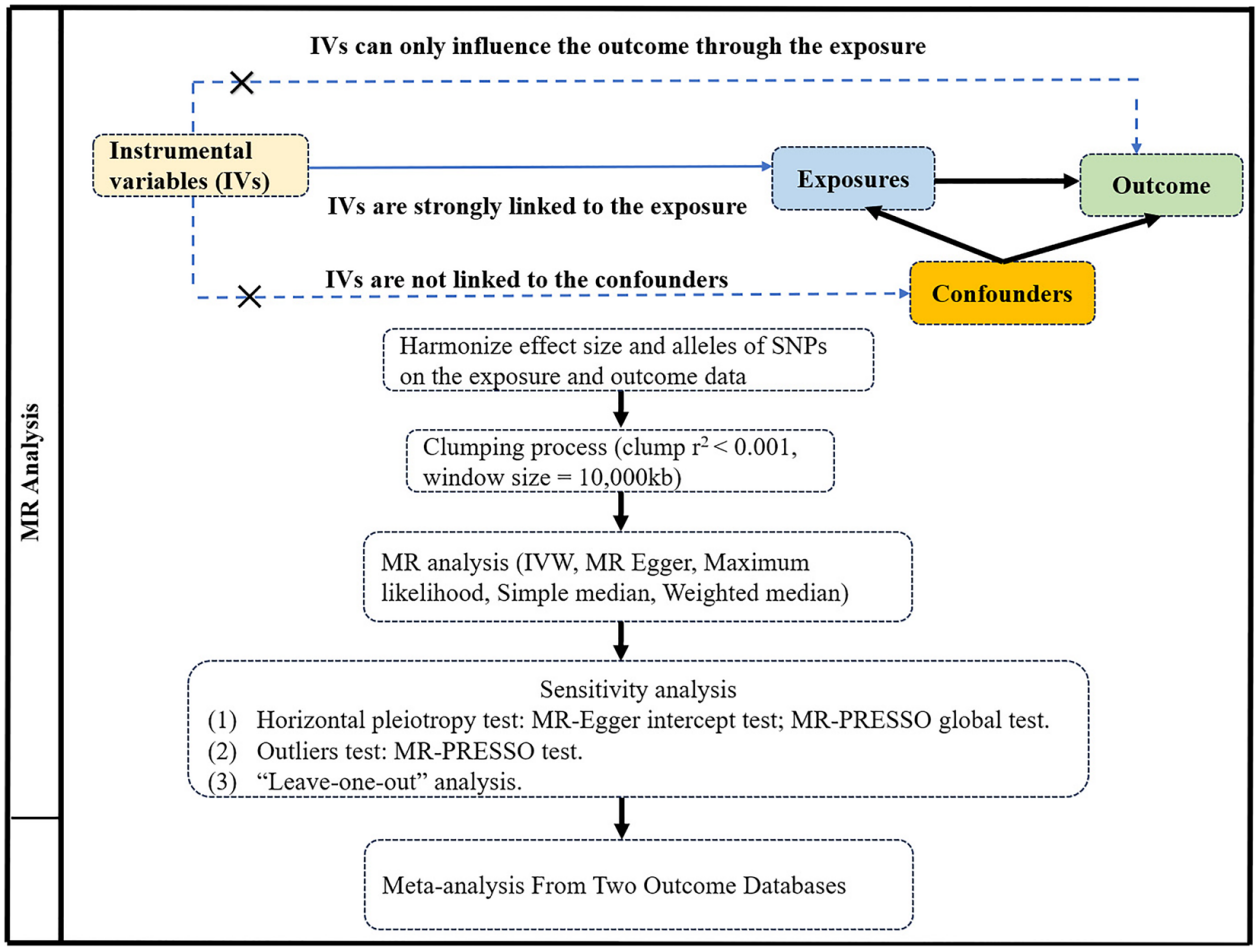
Flowchart of a MR study.

Mendelian randomization (MR) analyses employ genetic variation as an instrumental variable to mitigate the influence of confounding factors^14^. This study utilized MR analysis to evaluate the genetic association and causal association between metabolic syndrome (MetS), its constituents, and PCa derived from data in the largest aggregated genome-wide association study (GWAS). Due to the fact that genotypes are established prenatally and randomly assigned during meiosis^15^, MR analyses prove to be effective in reducing confounding factors and ascertaining the association between exposure and outcome. In MR analyses, the instrumental variable (IV) must fulfill three fundamental assumptions^16^ (1) the instrumental variable for genetic variation must exhibit a strong association with the exposure; (2) no associations were allowed between the genetic instrumental variables and all confounders; and (3) The genetic instrumental variable and the outcome should not share an identical cause, influencing the outcome solely according to the exposure variable (Fig. 1).

### Genetic instrument variable selection

The data for this study were obtained from the latest summarized data of genome-wide association studies (GWAS). Several criteria were established for data processing: (1) We selected the SNP for exposure at *p* < 5 × 10^−8^; (2) linkage disequilibrium effects were controlled using the PLINK clustering method, with an LD r^2^ threshold of < 0.001 and a clustering window of 10,000 kb; (3) SNPs linked to confounding factors were excluded; (4) ambiguous and palindromic SNPs were excluded through coordinated processing; and (5) SNPs exhibiting pleiotropy were eliminated^17^.

### Source of data

The Metabolic Syndrome Genetic Tool utilized the most recent data from the Complex Trait Genetics Laboratory (CTG), which comprised 461,920 validated individuals of European ancestry^18^. After data processing, 155 SNPs were incorporated into the Mendelian randomization analysis. The waist circumference (WC) data were sourced from the Genetic Investigation of Anthropometric Traits (GIANT) consortium, which provided GWAS summary data from 224,459 subjects (142,762 Europeans)^19^. Blood pressure (BP) data were extracted from the GWAS summary data of systolic blood pressure (SBP) and diastolic blood pressure (DBP) provided by the International Blood Pressure Alliance, including 757,601 individuals of European descent^20^. GWAS data on lipid traits, triglycerides (TG) and high-density lipoproteins (HDL) were obtained from the Global Lipid Genetics Consortium, which consisted of 188,577 individuals, with 95% being of European ancestry^21^. Fasting blood glucose (FBG) data originated in the Meta-analysis of the Glucose and Insulin-Related Traits Consortium, comprising 281,416 subjects, with over70% being of European descent^22^.

Data on genetic variants in PCa was acquired from the PRACTICAL consortium, which conducted a large study including 79,148 cases and 61,106 controls of European ancestry, ultimately including 20,346,368 SNPs^23^. Not only that, we also included the GWAS data from the FinnGen consortium for analyses, which included 151,99 cases and 131,266 controls^24^. For more information on the cohorts, genotypes, outcome criteria and association tests used, please visit the FinnGen web page (https://www.finngen.fi/en) and the PRACTICAL Consortium. Detailed descriptions of the exposure and outcome factors are given in Table 1. The above data are derived from publicly available databases and do not require additional ethical applications.

**Table 1:** Sources of phenotypic descriptive statistics for inclusion in genome-wide association studies of exposures and outcomes.

	PMID	Samples	Consortium or cohorts	Source
MetS	35,983,957	461,902	NA	https://ctg.cncr.nl/software/
WC	25,673,412	224,459	ANthropometric Traits (GIANT) consortium	https://gwas.mrcieu.ac.uk/
SBP	30,224,653	757,601	International Consortium of Blood Pressure (ICBC)	https://gwas.mrcieu.ac.uk/
DBP	30,224,653	757,601	International Consortium of Blood Pressure (ICBC)	https://gwas.mrcieu.ac.uk/
TG	24,097,068	188,577	Global Lipids Genetics Consortium (GLGC)	http://lipidgenetics.org/
HDL	24,097,068	187,167	Global Lipids Genetics Consortium (GLGC)	http://lipidgenetics.org/
FBG	34,059,833	281,416	The Meta‐Analyses of Glucose and Insulin‐related traits Consortium (MAGIC)	https://magicinvestigators.org/
Prostate cancer	29,892,016	79,148/61,106	PRACTICAL	https://gwas.mrcieu.ac.uk/
Prostate cancer	NA	151,99/131,266	FinnGen	https://r10.finngen.fi/

*MetS* metabolic syndrome, *WC* waist circumference, *SBP* systolic blood pressure, *DBP* diastolic blood pressure, *TG* triglycerides, *HDL* high density lipoprotein FBG fasting blood glucose.

### Statistical analysis

In our study, we employed inverse variance weighting (IVW), MR-Egger regression, and weighted median as the main approach of analysis. These methods can help us evaluate the causality between MetS and PCa. The IVW method was served as one of the main analytical approach due to its ability to generate robust causal estimates while accounting for pleiotropy^25^. To evaluate the resilience of the IVW results, sensitivity analyses were performed using MR-Egger regression and weighted median^26,27^. In addition to this, to increase the credibility of our findings, we conducted Cochran's Q tests and MR-Egger intercept experiments to investigate heterogeneity and horizontal pleiotropy across all SNPs^27,28^, and applied MR-PRESSO to identify and exclude outliers^29^. We use PhenoScanner to filter and remove confounding factors.

From the results of previous studies, we found that smoking and alcohol consumption may be risk factors for PCa and may influence the effect of MetS on PCa. To rule this out we performed MVMR to correct for these confounders. The GWAS data sources for smoking and alcohol consumption are shown in Table 2.

**Table 2:** Details for GWAS of smoking and alcohol consumption.

Mediators	Sample size	GWAS ID
Smoking	462,434	ukb-b-223
Alcohol consumption	462,346	ukb-b-5779

All statistical analyses were conducted using the “TwoSampleMR” package (version 0.5.6) in R software (version 4.3.1). *P* < 0.05 was considered statistically significant.

## Result

### Univariable MR

After rigorous screening of all the data, the detailed summary data can be found in Supplementary Material1. All genetic instrumental variables had F values exceeding 10, suggests that instrumental variables for the metabolic syndrome and its components can better avoid bias from potentially weak instrumental variables. The result of PCa data from the PRACTICAL Consortium showed a negative causal association between MetS and PCa (OR = 0.842, 95%[CI] = 0.766–0.926, *p* = 0.0004). However, the result of MR analysis from the FinnGen consortium database showed no association between MetS and PCa (OR = 1.09, 95%[CI] = 0.929–1.279, *p* = 0.29). Finally, we used meta-analysis to determine the overall causality, and the result suggested that there was no causal relationship between MetS and PCa (OR = 0.95, 95%[CI] = 0.738–1.223, *p* = 0.691).

**Figure 2:**
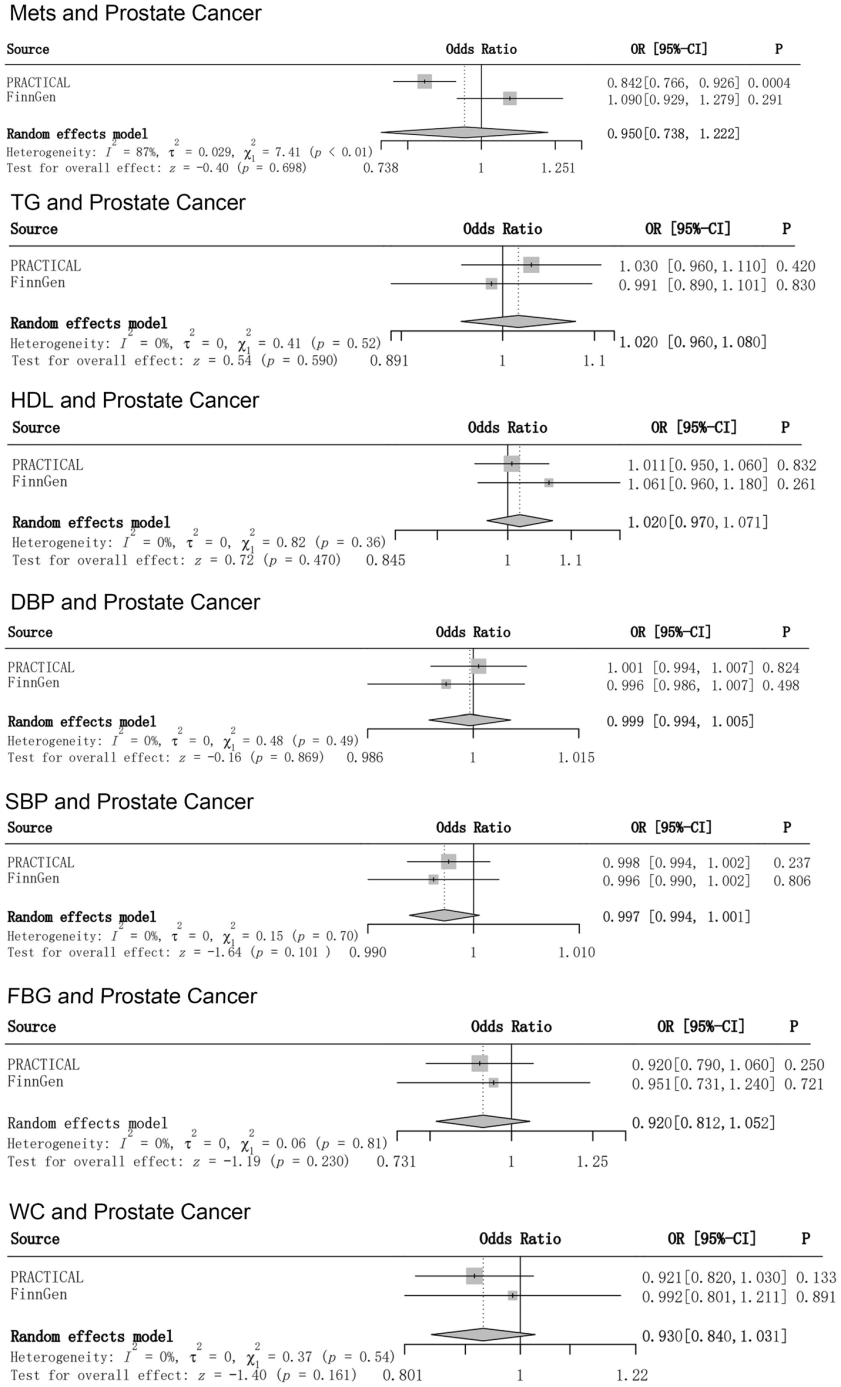
Risk relationship between MetS and its components and PCa.

For the components of the metabolic syndrome, results from all databases and meta-analyses suggested no causal association with PCa. For detailed results, please see Fig. 2. Although our Q-test results showed partial heterogeneity. However, all MR-Egger regressions exhibited no signs of potential horizontal pleiotropy (all *p*-values for intercepts > 0.1) (Table 3), and the PhenoScanner, which we employed to avoid potential pleiotropic effects due to confounders, did not find genetic instrumental variables associated with other phenotypes. Detailed SNP data are available in Supplementary Material 2.

**Table 3:** Details of heterogeneity and pleiotropy check in the MR analysis.

Exposure	Outcome date source	Heterogeneity				Horizontal pleiotropy	
		IVW		MR egger			
		Q	*P*	Q	*P*	Q	*P*
Mets	PRACTICAL	208.774	0.002	208.565	0.0019	0.000948	0.696
	FinnGen	321.798	1.85E−09	321.708	1.42E−09	− 0.000911	0.821
TG	PRACTICAL	51.122	0.112	46.906	0.180	0.000549	0.068
	FinnGen	85.278	0.003	85.238	0.002	0.000659	0.877
HDL	PRACTICAL	97.211	0.021	96.411	0.02	0.001867	0.449
	FinnGen	172.778	1.95E−08	170.861	2.24E−08	− 0.00446	0.343
DBP	PRACTICAL	684.105	4.66E−15	683.265	4.28E−15	0.001071	0.474
	FinnGen	713.485	4.48E−17	712.969	3.82E−17	0.001347	0.58
SBP	PRACTICAL	667.819	1.39E−13	667.396	1.19E−13	− 0.000778	0.606
	FinnGen	652.694	7.04E−12	652.509	5.84E−12	0.000832	0.729
FBG	PRACTICAL	27.246	0.293	27.234	0.246	0.000443	0.921
	FinnGen	46.672	0.027	42.411	0.052	0.01249	0.098
WC	PRACTICAL	41.249	0.183	40.641	0.169	0.003084	0.487
	FinnGen	77.467	0.001	77.08	0.001	− 0.003679	0.649

*MetS* metabolic syndrome, *WC* waist circumference, *SBP* systolic blood pressure, *DBP* diastolic blood pressure, *TG* triglycerides, *HDL* high density lipoprotein, *FBG* fasting blood glucose.

### MVMR

In the MVMR analysis, after correcting for smoking and alcohol consumption, the IVW results of MVMR were consistent with the results of the univariable Mendelian randomization analysis, indicating that the conclusion of our study was not affected by the confounding factors of smoking and alcohol consumption. (Table 4).

**Table 4:** Association of MetS and Alcohol with PCa risk in MVMR.

adjust	Outcome date source						
	PRACTICAL				FinnGen		
	SNPs	OR (95%)	*p*		SNPs	OR (95%)	*p*
Smoking	178	0.846(0.761–0.94)	0.002		185	1.061(0.894–1.259)	0.495
Alcohol consumption	175	0.839(0.746–0.943)	0.003		183	1.012(0.841–1.219)	0.899

## Discussion

This study investigated the causal relationship between the metabolic syndrome and its components and PCa using MR analyses. Although the result of PCa data from the PRACTICAL Consortium showed a negative causal association between metabolic syndrome and PCa (OR = 0.842, 95%[CI] = 0.766–0.926, *p* = 0.0004), these were considered to be serendipitous findings, as no significant causal relationships were found in the FinnGen Consortium database (OR = 1.09, 95%[CI] = 0.929–1.279, *p* = 0.29) and meta-analyses (OR = 0.95, 95%[CI] = 0.738–1.223, *p* = 0.691). In summary, we found no valid evidence to support a causal relationship between metabolic syndrome and its components and PCa. We conducted a pleiotropy check, which suggests that the likelihood of horizontal pleiotropy exerting an influence on our findings is minimal.

PCa is one of the most frequently detected cancers in men, with statistics about 1.4 million new cases reported in the 2020 global epidemiological survey^30^. PCa is particularly common in older men^31^. Metabolic syndrome is becoming more prevalent in the population due to the increase in high-calorie, low-fiber diets and the decrease in physical activity due to mechanized transport and sedentary leisure practices^5^. In addition to this, Metabolic abnormalities frequently coexist in the elderly and are intricately related to age^32^. Whether a causal link exists in PCa and metabolic syndrome has caught the attention of researchers. There have been studies that have begun to explore the link between the two diseases. A cohort study found that metabolic syndrome appears to act as a potential risk factor for PCa, and they concluded that lipid and cholesterol levels are the main factors that influence whether PCa occurs or not^7^. However, it is difficult for such cohort studies to investigate the causal relationship between metabolic syndrome and PCa because of subject recall bias, and also because the information collected is often not comprehensive enough, and finally, other relevant confounding factors may also have an impact on the results of the study^33^. In a meta-analysis examining the association between metabolic syndrome and its components with PCa, K Esposito et al. concluded that metabolic syndrome elevates the likelihood of developing PCa by 12 per cent, and for the components of the metabolic syndrome, only hypertension and waist circumference of more than 102 cm increase the risk of PCa by 15 per cent and 56 per cent, respectively^34^. It is worth noting that meta-analyses can only summaries the results of observational studies and cannot effectively demonstrate causality, while differences in the methodology of the original studies and publication bias make the results of meta-analyses potentially compromised^35^. However, studies have also concluded that the metabolic syndrome reduces the risk of PCa, and a cohort study by Aaron J. Tande et al. concluded that the metabolic syndrome was linked to a decreased incidence of PCa, and that the negative association between the metabolic syndrome and the incidence of PCa strengthened when diabetes was excluded^8^. While cohort studies can clarify exposure and subsequent outcomes and help determine the causal relationship between exposure and outcome, it cannot be ignored that cohort studies are prone to lost tracking and elimination rates, which may affect the credibility of the findings^36,37^. In addition to these, a retrospective cohort study in China explored whether metabolic syndrome affects PCa recurrence after surgery^38^. They included 214 PCa patients who underwent radical prostatectomy, and ultimately also concluded that there was absence of causal link between metabolic syndrome and PCa recurrence. Results similar to that, the outcomes of the EPICAP case–control study conducted by Céline Lavalette et al. showed no association between metabolic syndrome and PCa^9^. Furthermore, their point of view suggest that the usage of therapeutic non-steroidal anti-inflammatory drugs (NSAIDs) modifies the risk relationship between MetS and PCa^9^. These findings are consistent with our results. However, retrospective case–control studies can lead to confounding of findings because of recall bias and selection bias, as well as other potential confounders that are difficult to control for^39,40^. Most importantly, it is often difficult to establish a causal relationship between exposure and outcome because it is not possible to accurately determine whether the exposure occurred before or after the disease^41^. Different conclusions were reached for different studies. In light of the divergent conclusions reached by various studies, we speculated whether there is a causal association between metabolic syndrome and PCa, and we analyzed this using Mendelian randomization studies. In conclusion, we concluded that there is no causal link between the metabolic syndrome and its components and PCa based on our results.

The development of PCa has been closely linked to testosterone levels. Some studies suggest that individuals with high testosterone levels are more likely to develop PCa. Katherine S Ruth et al.^42^, working with human genes, found that higher testosterone was detrimental to a man's prostate, and their Mendelian randomization analyses showed that elevated testosterone levels increased a man's risk of PCa by 23% per one standard deviation increase. However, there is no consensus on whether people with metabolic syndrome have higher or lower levels of testosterone than normal people.

For the constituents of the metabolic syndrome, although our results do not indicate a causal relationship between them, there has also been literature that concludes a link between other components of the metabolic syndrome and PCa. A systematic review by Danielle Crawley et al. concluded that type 2 diabetes is a protective factor for PCa ^43^. The meta-analysis by S Bonovas et al. included 14 studies, comprising both case–control and cohort designs, and ultimately concluded an inverse relationship between diabetes and PCa^44^. This study aims to explore the risk factors of PCa, thereby aiding in the identification of high-risk individuals and improving the efficiency of PCa screening and early detection opportunities. Furthermore, we hypothesize that treatments targeting metabolic syndrome, such as lifestyle interventions and pharmacotherapy, may contribute to improving the prognosis of PCa patients^45^. This would represent a highly intriguing research avenue. However, based on our findings, further investigation into the relationship between metabolic syndrome and its components with PCa is warranted. For instance, whether this relationship remains consistent among Asian and African populations as observed in our study merits further exploration. In the next five years, we believe that more research teams and further research will be involved in the study to investigate the potential causal connection between metabolic syndrome and PCa, as both diseases have a high prevalence in the older population^46,47^.

### Strength and limitation

Our research has certain advantages. By using Mendelian analyses, we conducted an investigation into the causal relationship between metabolic syndrome and its components with PCa while minimizing the influence of confounders and reverse causation effects on the results. In addition to this, we used meta-analysis to improve the precision of Mendelian randomization.

However, our study has several limitations that deserve attention. First, we acknowledge that our study was conducted in a European population, therefore our findings may not be generalizable to other racial groups. Additionally, although we carefully selected instrumental variables, the complete elimination of all confounding variables remains unattainable. Third, our study was unable to detect the nonlinear causal relationship between metabolic syndrome and PCa. Lastly, we did not stratify metabolic syndrome by gender or age, which may have influenced our study results. Therefore, to further explore the association between metabolic syndrome and its components with PCa, it is necessary to obtain larger sample sizes from other ethnic populations for validation. Not to be overlooked, it is also important to conduct deeper analyses.

## Conclusion

We conducted a two-sample Mendelian randomization (MR) analysis to research the causal relationship between metabolic syndrome (MetS), its components, and PCa. Moreover, we chose the FinnGen database as the validation set. Although the results of the data from the PRACTICAL consortium indicate a negative causal association between metabolic syndrome and PCa, the validation set from the FinnGen database suggests that there is no causal association between the two. Finally, we used a meta-analysis to resolve this discrepant result and enhanced the precision of Mendelian randomization, ultimately there was no evidence of a causal relationship.. Therefore, we conclude that there is no significant causal relationship between metabolic syndrome and its components and PCa. Further research is required to explore the impact of MetS and its components on PCa in diverse populations.

### Supplementary Information

Supplementary Figures.

Supplementary Information.

## Data Availability

All datasets in this study are available for download in the online dataset and further contact the corresponding author if necessary.
