# Supplementary material for: A Mendelian randomization study between metabolic syndrome and its components with prostate cancer
Source: Sci Rep. 2024 Jun 21;14:14338. doi: 10.1038/s41598-024-65310-y (PMC11192917; doi:10.1038/s41598-024-65310-y)
Supplement: Supplementary file 1 — Supplementary Figures. [file 41598_2024_65310_MOESM1_ESM.pdf]

Supplementary Material

A study of the causal association between metabolic syndrome and its components with prostate cancer: a Mendelian randomization analysis

Long Xia[†], Xiao-dong Yu[†], Li Wang[†], Lin Yang, Er-hao Bao, Ben Wang, Ping-yu Zhu[*]
*Corresponding author: Ping-yu Zhu, Email: zhupingyu@nsmc.edu.cn

1.Supplementary Figures

A: metabolic syndrome (MetS); **B**: triglycerides(TG); **C**:high density lipoprotein(HDL); **D**:diastolic blood pressure(DBP);**E**:systolic blood pressure (SBP); **F**:fasting blood glucose(FBG); **G**:waist circumference(WC)

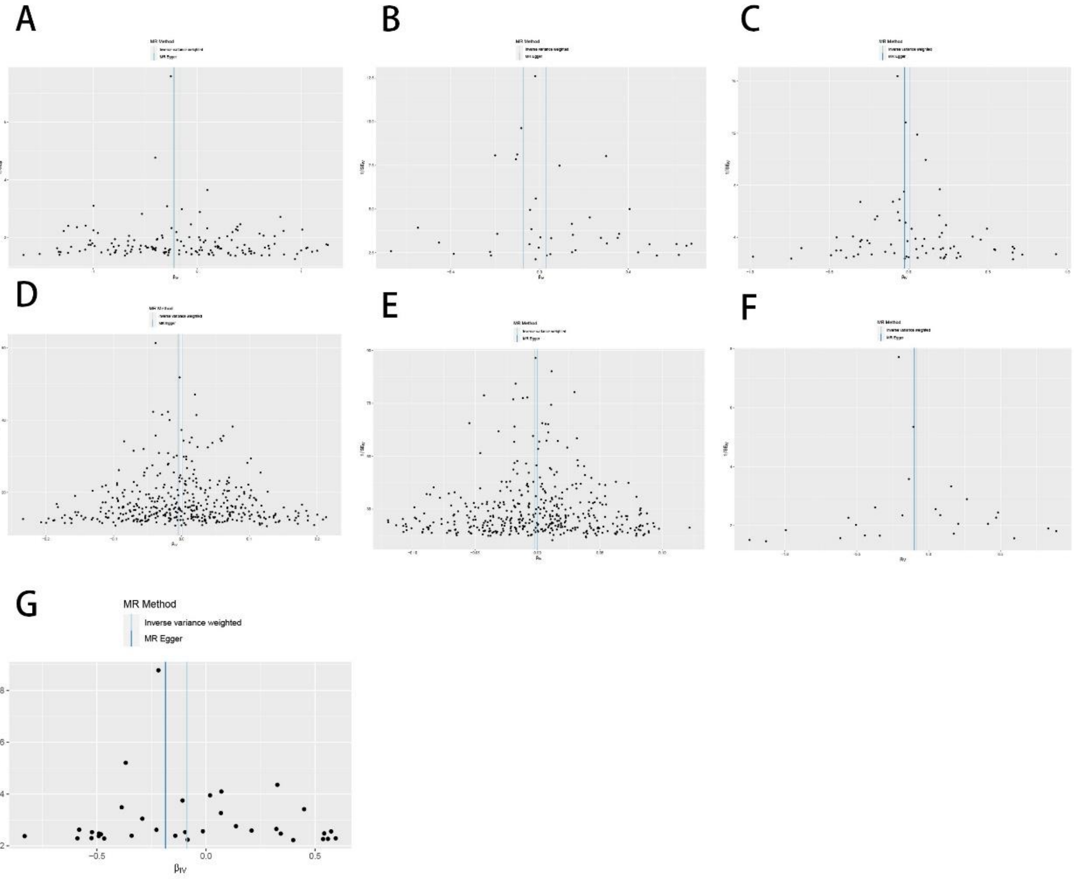

Supplementary Figure 1. Funnel plot analysis of the metabolic syndrome and its components in relation to prostate cancer (outcome data from the PRACTICAL Consortium)

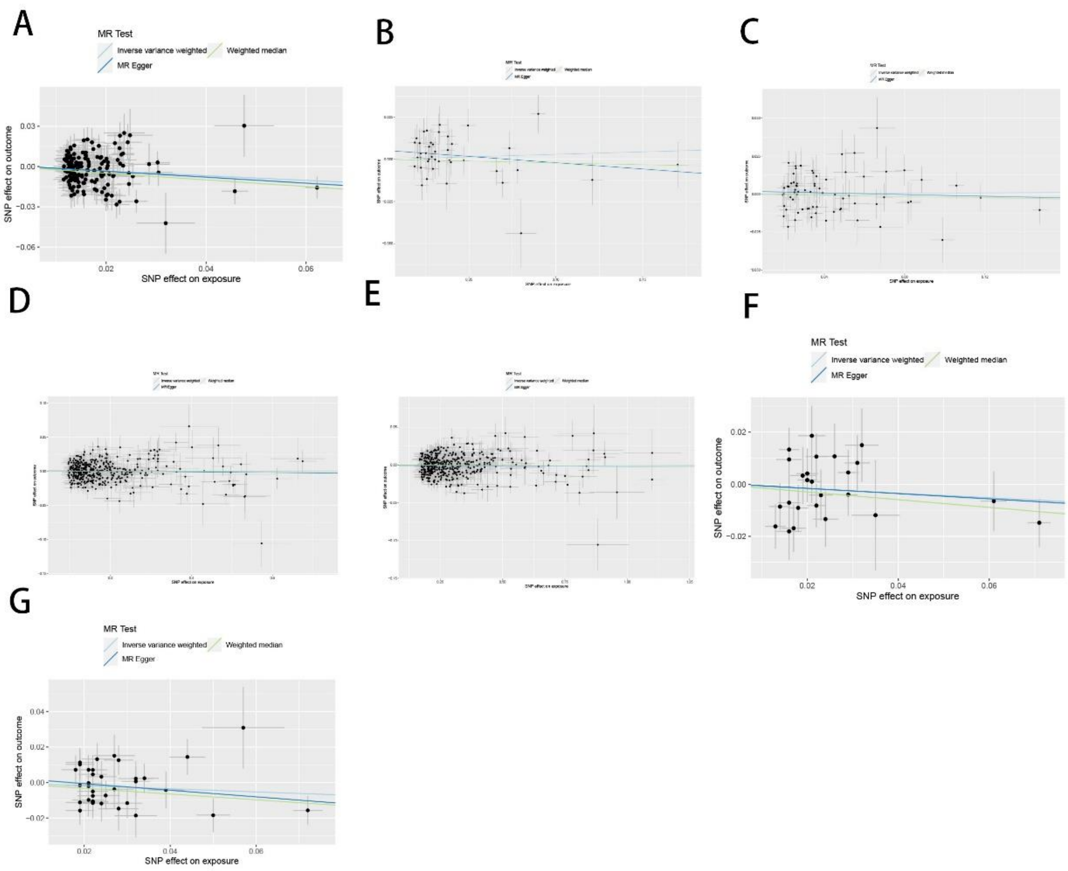

Supplementary Figure 2. Scatterplot analysis of the metabolic syndrome and its components in relation to prostate cancer (outcome data from the PRACTICAL Consortium)

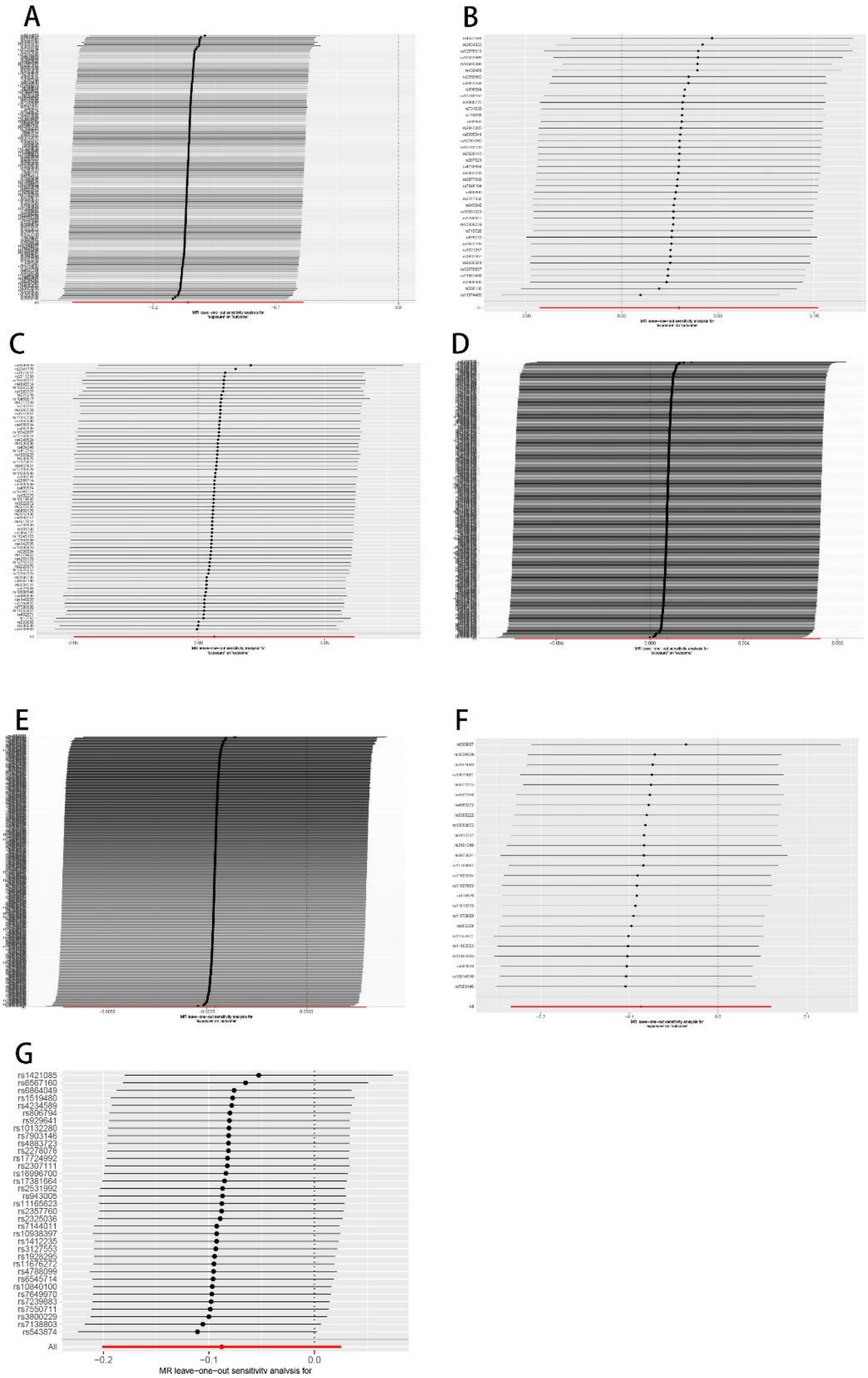

Supplementary Figure 3. Leave-one-plot analysis of the metabolic syndrome and its components in relation to prostate cancer (outcome data from the PRACTICAL Consortium)

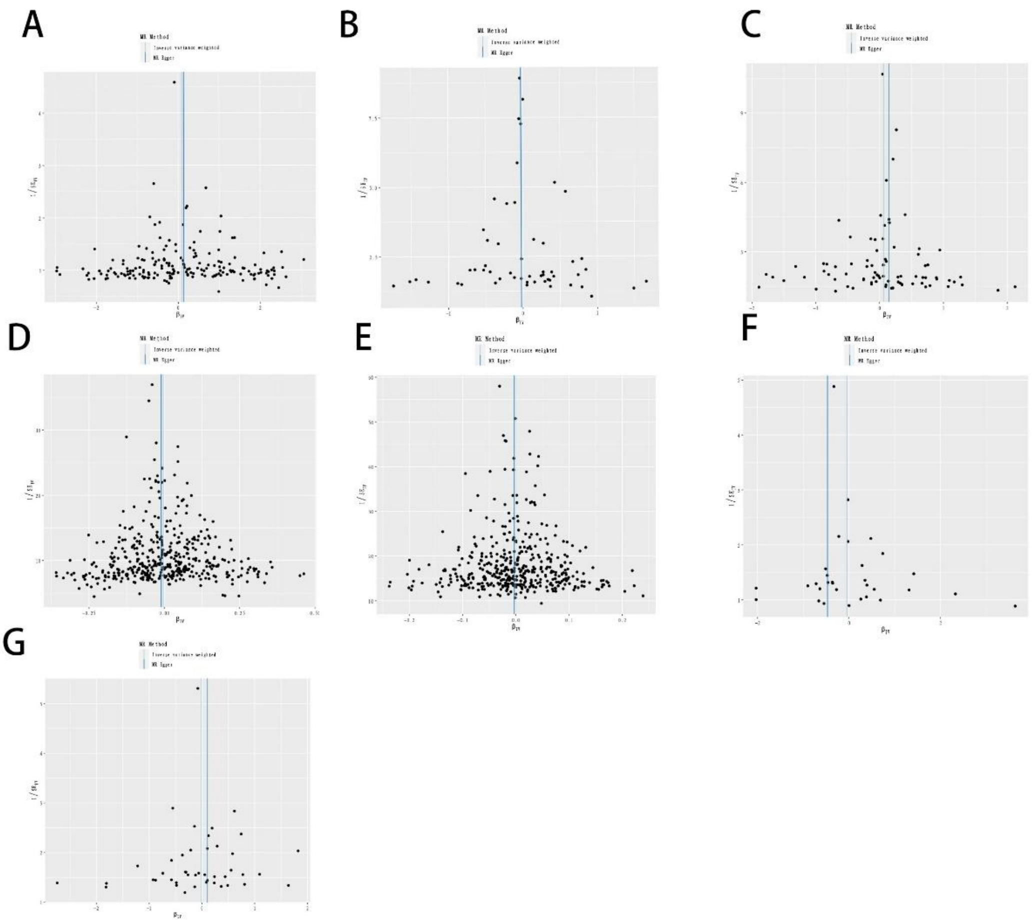

Supplementary Figure 4. Funnel plot analysis of metabolic syndrome and its components in relation to prostate cancer (outcome data from FinnGen Consortium)

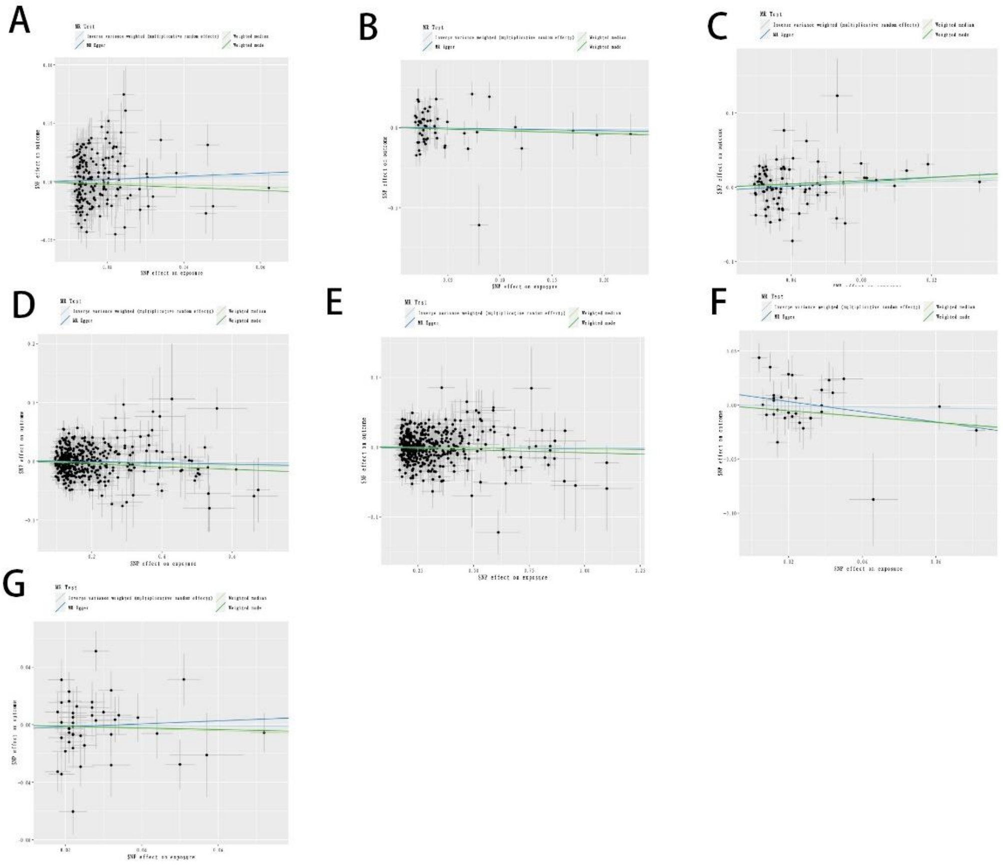

Supplementary Figure 5. Scatterplot analysis of the metabolic syndrome and its components in relation to prostate cancer (outcome data from the FinnGen Consortium)

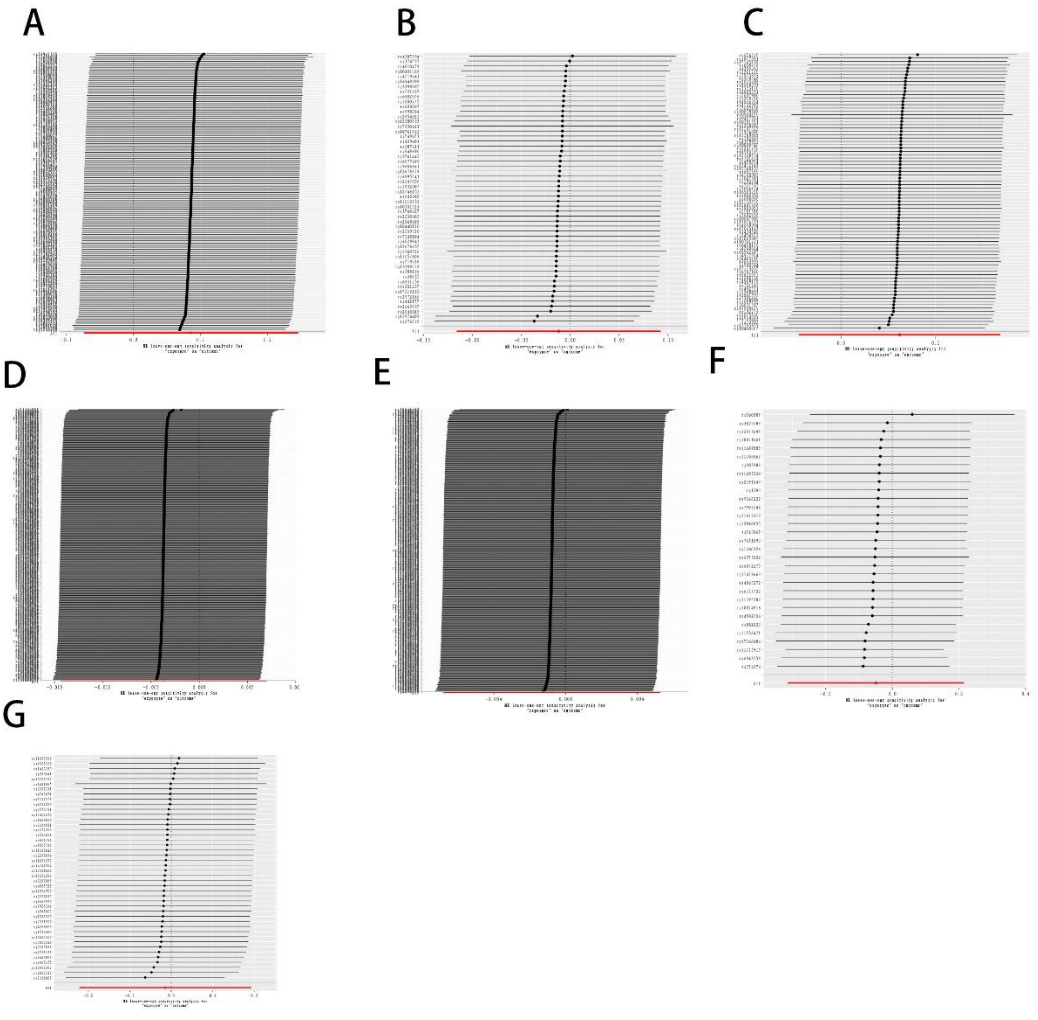

Supplementary Figure 6. Leave-one-plot analysis of the metabolic syndrome and its components in relation to prostate cancer (outcome data from the FinnGen Consortium)
